# Molekularpathologie in der Behandlung des Lungenkarzinoms – interdisziplinärer Blick auf die thoraxchirurgische Bedeutung

**DOI:** 10.1007/s00104-021-01544-0

**Published:** 2021-12-14

**Authors:** Sebastian Krämer, Hubert Wirtz

**Affiliations:** 1 Klinik und Poliklinik für Viszeral‑, Transplantations‑, Thorax- und Gefäßchirurgie, Bereich Thoraxchirurgie, Universitätsklinikum Leipzig AöR, Liebigstr. 20, Haus 4, 04103 Leipzig, Deutschland; 2 Klinik für Onkologie, Gastroenterologie, Hepatologie, Pneumologie und Infektiologie, Bereich Pneumologie, Universitätsklinikum Leipzig AöR, Leipzig, Deutschland

**Keywords:** Zielgerichtete Therapie, Adjuvante Therapie, Epidermal-growth-factor-Rezeptor, Next generation sequencing, Salvage-Chirurgie, Targeted treatment, Adjuvant therapy, Epidermal growth factor receptor, Next generation sequencing, Salvage surgery

## Abstract

Die Therapie des Lungenkarzinoms wurde in den letzten Jahren zunehmend durch die Etablierung tumorspezifisch zielgerichteter Pharmaka und immunmodulatorischer Ansätze ergänzt und hat dadurch rasant an Komplexität gewonnen. Bessere Überlebensdaten und Erkenntnisse über andere Nebenwirkungensspektren und Rezidivcharakteristika begleiten diese Entwicklung. Dem Kliniker verlangt diese Entwicklung eine stete Wachsamkeit in der Stratifizierung der Behandlungsoptionen ab. Dieser Artikel gibt einen Überblick über die klinisch aktuell relevanten Ansätze der „targeted therapies“ in der Behandlung des Lungenkarzinoms und stellt Verbindungen zur Thoraxchirurgie dar. Mit der Darstellung der Optionen einer zielgerichteter Therapie wird beantwortet, welche Rolle sie in der adjuvanten Therapie bei nachgewiesener Mutation des Epidermal-growth-factor-Rezeptors (*EGFR*) spielen, wann eine Salvage-Operation infrage kommt und wie durch die „targeted therapies“ in Einzelfällen ein kuratives Therapiekonzept erarbeitet werden kann. Jedes Lungenkarzinom verlangt ab dem frühestmöglichen Zeitpunkt in der Diagnosefindung nach einer molekularen Analyse auf therapierelevante Mutationsmuster. Interdisziplinäre Konzepte können individualisiert das Langzeitüberleben des Patienten gewährleisten.

## Abstract (en)

Treatment modalities of lung cancer have rapidly evolved in recent years by the establishment of tumor-specific targeted drugs and immunomodulatory concepts and the complexity has rapidly increased. This development is accompanied by improved survival data and knowledge of other spectra of side effects and recurrence characteristics. This development requires that clinicians maintain a constant vigilance in the stratification of treatment options. This article gives an overview of the current clinically relevant approaches of targeted treatment of lung cancer and points out possible links to thoracic surgery. The presentation of the options of targeted therapy demonstrates which role they play in the adjuvant treatment in cases of proven mutations of epidermal growth factor receptor (*EGFR*), when a salvage operation can be used and how a curative treatment concept can be elaborated in individual cases through targeted treatment. Every lung cancer ultimately requires a molecular analysis of treatment-relevant mutation patterns at the earliest possible time in the diagnostics. Interdisciplinary concepts can individually guarantee the long-term survival of the patient.

## Hintergrund

Die Therapie des Lungenkarzinoms hat sich in den vergangenen 10 Jahren sehr stark verändert, indem molekularpathologische Erkenntnisse zunehmend in die Therapieentscheidungen eingeflossen sind. Diese Entwicklung führte zu einem immer differenzierteren und zunehmend individualisierten Vorgehen in der Behandlung der weiterhin weltweit am häufigsten vorkommenden und immer noch mit der höchsten Mortalität verbundenen Krebserkrankung [1]. Epidemiologische Daten lassen Rückschlüsse auf eine Steigerung des auf das „non-small-cell lung cancer“ (NSCLC) bezogene Überleben zu, die nicht allein durch eine moderate Reduktion der Inzidenz, sondern auch durch die Erfolge differenzierterer Therapien, insbesondere der „targeted therapies“ zu erklären sind [2]. Gleiches gilt nicht für das kleinzellige Lungenkarzinom (SCLC), für welches bis dato keine zielgerichteten Therapien zur Verfügung stehen.

In der Klassifikation des Lungenkarzinoms gilt weiterhin die grundsätzliche Einteilung nach kleinzelligen und nichtkleinzelligen Karzinomen. Das nichtkleinzellige Lungenkarzinom wird histologisch weiter differenziert; hier sind vor allem die häufigen Vertreter der Adenokarzinome und mit geringerer Häufigkeit die Plattenepithelkarzinome vor anderen selteneren Varianten zu nennen. Für die Adenokarzinome wurde 2011 eine aktualisierte Einteilung durch die International Association for the Study of Lung Cancer (IASLC) veröffentlicht [3].

Trotz gleicher histologischer Subtypisierung ist allerdings inzwischen gut bekannt, dass es definierte genetische Veränderungen/Mutationen in den Tumorzellen gibt, die im Sinne von „Treibern“ das Wachstum dieser Zellen so maßgeblich bestimmen, dass ihre Blockade den klinischen Verlauf erheblich beeinflussen kann. Durch die neuen und schnellen Möglichkeiten zur Aufklärung der genetischen Information und ihrer individuellen Mutationen, z. B. mithilfe des „next generation sequencing“ (NGS), gelangen zunehmend mehr Mutationen in den Fokus, wenngleich noch nicht für alle ein entsprechendes Medikament zur Verfügung steht [4]. Zur Festlegung der individualisierten Therapie gehört daher heute neben der histologischen Typisierung und dem Ausbreitungsgrad eine molekulare Charakterisierung, die auch Eingang in die Leitlinienempfehlung gefunden hat [5]. Diese kann entweder mit dem Einzelnachweis von Genmutationen an bekannten Lokalisationen erfolgen oder mittels der schon erwähnten NGS-Technik. Letztere wird seit 2020 von der European Society of Medical Oncology (ESMO) zumindest für die Adenokarzinome der Lunge empfohlen, da sie besonders häufige und besonders variantenreiche Mutationen tragen können [6].

Nicht alle wesentlichen Veränderungen, die das Tumorwachstum treiben, sind einfache Veränderungen der genetischen Sequenz. Manche sind komplexe Translokationen, d. h. Repositionierungen genetischer Abschnitte mit Änderung der chromosomalen Struktur. Daraus beispielhaft entstandene daueraktivierende Genabschnitte bewirken nur selten, aktivierende Gene auch tatsächlich dauerhaft aktiv werden zu lassen.

## Mutationen von klinischer Relevanz

In der molekularen Diagnostik des Adenokarzinoms der Lunge werden aktuell das Vorliegen der folgenden genetischen Aberrationen geprüft [7].

- *EGFR*(„epidermal growth factor receptor“)-Exon-18–21-Mutationen,
- *ALK*(„anaplastic lymphoma kinase“)-Translokationen,
- *ROS-proto-oncogene-1-*Translokationen,
- *BRAF*(„B-rapidly accelerated fibrosarcoma“)-V600-Mutationen,
- *NTRK*(neurotrophe Tyrosinkinase)-Fusionsgene.

Zunehmend werden die folgenden Veränderungen mit aufgenommen:

- *BRAF*-NonV600-Mutationen,
- *HER2-*Amplifikationen,
- *KRAS*(„Kirsten rat sarcoma virus“)-Mutationen,
- *c‑MET*(„mesenchymal-epithelial transition“)-Alterationen mit *c‑MET-*Exon-14-Skipping-Mutationen, Amplifikation und Fusionen,
- *NRG1-*Gen-Fusionen,
- *RET*-Translokationen u. a.

Für das kleinzellige Karzinom und die Gruppe der Plattenepithelkarzinome liegen zwar Erkenntnisse über das Mutationsmuster vor; eine klinische Relevanz ergibt sich durch das Fehlen eines klinisch-therapeutischen Ansatzes zum aktuellen Zeitpunkt noch nicht [8, 9].

Die üblichen Behandlungsoptionen und -kombinationen der Chirurgie, Radiotherapie und Chemotherapie wurden über die Zeit durch immunmodulatorische und „targeted therapies“ ergänzt. In den komplexer werdenden Fragestellungen ergeben sich Lösungsansätze, die nur durch das Auseinandersetzen mit dem steten globalen Wissenszuwachs und der aktuelleren Studienlage sowie durch das Bewusstsein um die möglichen zum Einsatz kommenden Substanzen zu finden sind.

Auf die sich parallel entwickelnden und ergänzenden Therapieoptionen und -konzepte im Einsatz immunmodulatorischer Medikamente wird hier an dieser Stelle nicht eingegangen.

Repräsentativ werden im Folgenden vier Vertreter der oben genannten Mutationen näher beschrieben.

### Epidermal-growth-factor-Rezeptor

Der EGF-Rezeptor ist ein Protein der Wirbeltiere, welches sich als Transmembranrezeptor mit intrazytoplasmatischer Tyrosinkinasedomäne darstellt. Unter den pulmonalen Adenokarzinomen findet man eine Mutation des *EGFR* in den Ländern der sog. westlichen Zivilisation bei ca. 19 % aller NSCLCs. In den asiatischen Ländern liegt diese bei ca. 48 % der Lungenadenokarzinome vor [10].

Alle Adenokarzinome der Lunge sollten auf *EGFR*-Mutationen getestet werden, wenngleich diese am häufigsten bei Nichtrauchern, Frauen und Patienten jüngeren Alters gefunden werden. Lungenmalignome mit histologischer Mischkomponente, sofern sie einen Adenokarzinomanteil in sich tragen, sollten ebenfalls auf *EGFR*-Aberrationen untersucht werden.

Eine etablierte Möglichkeit der Testung mittels „liquid biopsy“ ist zwar weniger sensibel als eine Untersuchung am Biopsiematerial, zeigt aber eine hohe Spezifität.

**Figure 1:**
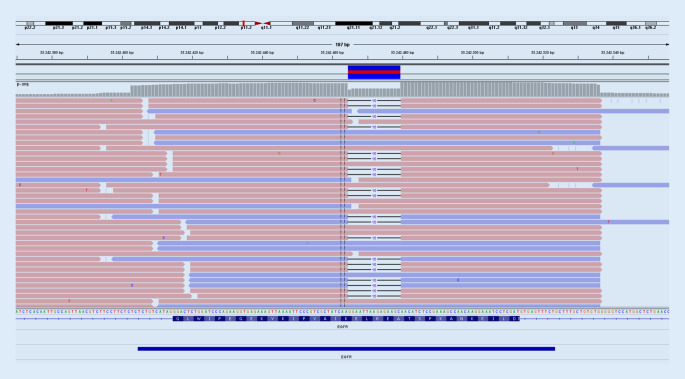

Die häufigen Deletionen im Exon 19 (Beispiel in Abb. 1) und die Punktmutation in Exon 21 (L858R) repräsentieren 85–90 % aller auf *EGFR*-Mutationen untersuchten Lungenkarzinome [11]. Diese sprechen auf die etablierten Tyrosinkinaseinhibitoren (TKI) an. So kann ein verbessertes Ansprechen des Tumors, ein verlängertes progressionsfreies Überleben und eine geringere Toxizität gegenüber der Standardchemotherapie erreicht werden. Das Gesamtüberleben ändert sich im Vergleich zu den klassischen Chemotherapeutika nicht. Die Auswahl des entsprechenden Präparats richtet sich nach dem Mutationstypus, dem erwarteten Nebenwirkungsprofil und eventuellen bekannten Resistenzmechanismen [7].

Weitere Mutationen in Exon 18 und 20 sind selten und zeigen ein geringeres bis fehlendes Ansprechen auf TKIs. Kombinationen mit anderen Treibermutationen außerhalb des *EGFR* sind eine Rarität.

Lungenkarzinompatienten mit einer *EGFR*-Mutation zeigen ein teils eindrucksvolles Ansprechen auf TKIs [12]. Mit einem Auftreten von Resistenzen ist innerhalb von 10 bis 16 Monaten zu rechnen [13]. In den allermeisten Fällen liegt dann eine weitere, in diesem Fall dann eine Resistenzmutation in Exon 20 vor.

### „B-rapidly accelerated fibrosarcoma“

Als *BRAF* bezeichnet man das Gen des BRAF-Proteins. Diese Serin-Threonin-Kinase interagiert in der MAP(„mitogen-activated protein“)-Kinase-Signalkaskade mit einem Effekt durch aktivierende Phosphorylierungen der „mitogen-activated protein kinase-kinase“ (MEK; [14]). *BRAF*-Mutationen sind in etwa 3 % der Lungenadenokarzinome zu detektieren und kommen häufiger bei aktiven oder ehemaligen Rauchern vor. Es sind sowohl aktivierende wie inhibierende Mutationen bekannt. In der Hälfte der Fälle liegt eine aktivierende V600E-Substitutionsmutation vor. Ein „overlapping“ mit anderen bekannten Mutationen des Lungenkarzinoms ist eine Seltenheit.

Eine Kombination der „targeted therapies“ aus Dabrafenib und Trametinib ist hier aktuell die Therapie der Wahl. Ein klinisches Ansprechen („overall response“) ist in 63 % der Fälle zu erreichen. Allerdings sind diese guten Ansprechraten ausschließlich für die V600E-Mutation des Lungenadenokarzinoms beschrieben; somit ist die erwähnte Medikation auch nur für diese zugelassen [15].

### Anaplastic-lymphoma-Kinase

Eine Veränderung im Gen der Anaplastic-lymphoma-Kinase (*ALK*) lässt sich bei 3–7 % der Patienten mit einem pulmonalen Adenokarzinom nachweisen [16]. Die häufigste Mutation liegt auf Chromosom 2 und verbindet das N‑terminale Ende des Gens *EML4* mit dem C‑terminalen Ende der Kinasedomäne der *ALK* mit einer daraus resultierenden anhaltenden Aktivierung der *ALK* (Gentranslokation oder Geninversion). Wiederum handelt es sich bei den Lungenadenokarzinomen mit nachgewiesener *ALK*-Mutation um eher junge Patienten ohne klassische Raucheranamnese. Es sind 4 Thyrosinkinaseinhibitoren in der Erstlinientherapie für *ALK*-Translokationen zugelassen.

### „ROS proto-oncogene 1“

„ROS proto-oncogene 1“ (*ROS1*), ein Rezeptor der Insulinrezeptorfamilie, wirkt als Membranprotein über eine Tyrosinkinaseaktivität [17]. Translokation und Deletionen können auf Geneebene zu einem fusionierten Protein mit verschiedenen Gegenstücken (z. B. CD74) führen, mit der Folge einer Daueraktivierung des Gens von *ROS1*. Unter den pulmonalen Adenokarzinomen lässt sich ein *ROS1*-Fusionsgen in etwa einem Prozent der Fälle nachweisen, mehrheitlich unter jungen und nie rauchenden Patienten. Auch hier kommen bei positivem Nachweis einer aktivierenden Mutation 2 TKIs als Erstlinienbehandlung infrage.

Über eine zielgerichtete Behandlungsoption sollte aufgrund der Komplexität und Dynamik der Möglichkeiten bei nachgewiesenen Mutationen stets nur in einem, im idealen Fall pulmoonkologisch-thoraxchirurgischen Tumorboard befunden werden.

## NSCLC-Mutationen und Thoraxchirurgie

Die alleinige Bedeutung von Mutationen unter den pulmonalen Nichtkleinzellern im kurativ intendiertem Stadium bis UICC (Union internationale contre le cancer) IIIA im Zusammenspiel mit den beiden assoziierten Fachrichtungen Thoraxchirurgie und Strahlentherapie ist zum jetzigen Zeitpunkt noch nicht abschließend zu interpretieren. Eine positive *EGFR*-Mutationen gilt als positiv prognostischer Faktor unter den chirurgisch resezierten pulmonalen Adenokarzinomen; allerdings nicht bei den Subtypen Exon-21-L858R-Mutation und die Exon-19-Deletion [18].

Die Nutzung der TKIs in der adjuvanten Therapie von *EGFR*-mutierten NSCLC wurde aktuell untersucht (83 % reduziertes Rezidiv- und Sterberisiko vs. Placebo im Stadium II+IIIA; Hazard Ratio [HR] = 0,17; 99,06 %-Konfidenzintervall [KI]: 0,11–0,26; *p* < 0,001; [19]). Bedingt durch den Vergleich gegen ein Placebo ist hier der eindeutige Nachweis eines positiven Effektes gegenüber der klassischen adjuvanten Chemotherapie offen, wenngleich ein solcher durch ein reduziertes Nebenwirkungsspektrum antizipiert werden kann (ADAURA ClinicalTrials.gov number, NCT02511106; [19]). Für *EGFR*-mutierte NSCLC mit einer Ex19del oder L858R-Mutation ist Osimertinib im Stadium IB/II/IIIA nach kurativ intendierter Resektion als adjuvante Therapie 2021 zugelassen worden.

Durch die Entwicklung der TKIs und deren systematische Anwendung gelingen teils hohe Ansprechraten, die wiederum zu Befundkonstellationen führen können, primär nichtresektable Lungenkarzinome nach Primärtherapie doch radikal resezieren zu können. Der Umstand der Nichtresektabilität bezieht sich diesbezüglich auf lokale Charakteristika, z. B. Größe oder Infiltrationstiefe des Tumors und vielmehr nicht auf das Metastasierungsmuster der Lymphknoten oder Fernmetastasen, wenngleich auch hier in absoluten Einzelfällen Konstellationen hin zur Resektion denkbar sind.

Eine chirurgische Option in der Behandlung primär nichtresektabler Stadium-III/IV-NSCLC ist die sog. Salvage-Resektion residueller Tumoren oder rekurrenter Läsionen nach einer definitiv geplanten Radio-/+Chemotherapie [20]. Zwei Arbeiten von Hishida et al. berichten von Stadium-IV-NSCLC-Patienten (*n* = 9) mit Tumorresektion nach vorheriger Gefitinib-Gabe. Als Indikationen wurden sowohl lokale Tumorpersistenz, Rekurrenz oder erneutes Wachstum nach erfolgter TKI-Therapie angesehen. Der Median im Gesamtüberleben war mit 32 Monaten bemerkenswert. Eine weitere Kleinserie (*n* = 4) der gleichen Arbeitsgruppe konnte trotz rekurrentem Verhalten des Tumors bei 3 der 4 Patienten ein Überleben aller Patienten von mindestens 4 Jahren zeigen [21, 22]. Auf Grundlage ungenügender Daten kann man die resezierende Chirurgie bei diesen Patienten als höchst individualisierte Behandlungsoption zumindest anerkennen. Ähnliches lässt ein Fallbericht eines Patienten mit *ALK*-mutiertem und TKI-vorbehandeltem NSCLC vermuten [23].

## Fazit

Die Entwicklung zielgerichteter Therapieoptionen in der Behandlung des „non-small-cell lung cancer“ (NSCLC) war ein wirklicher Paradigmenwechsel – zunächst in der Entwicklung eines erweiterten Verständnisses des Tumorverhaltens und im Weiteren in der Etablierung differenzierter Therapiekonzepte mit teils hervorragenden Überlebensdaten. Heute darf nicht nur keinem Patienten eine Testung auf Tumormutationen vorenthalten werden, es muss darüber hinaus vom Behandlungsteam eine stete Wachsamkeit eingefordert werden, sich mit den ständigen Entwicklungen und Neuerungen auf diesem Gebiet auseinanderzusetzen. Die Molekularpathologie (besonders im Hinblick auf „next generation sequencing“ und die „liquid biopsies“ zirkulierender DNA) und Pharmakologie sowie deren beider avisierte Weiterentwicklung werden den Rhythmus und die Geschwindigkeit vorgeben, mit der sich der Kliniker den pulmoonkologischen Herausforderungen stellen kann und muss. Der Stellenwert der Thoraxchirurgie in der Behandlung des NSCLC bleibt unbestritten; durch die „targeted therapies“ können in Einzelfällen kurative Therapiekonzepte erarbeitet werden.

Durch weitere neue Präparate und die Etablierung individualisierter Therapieregime in der Behandlung des Lungenkarzinoms besteht begründete Zuversicht, dass die positive Entwicklung der letzten Jahre unter unbedingtem Nutzen der Interdisziplinarität anhält [2]. Weitere Studien müssen in Zukunft die Rolle der Thoraxchirurgie in der Therapie „mutierter“ Bronchialkarzinome besser ausleuchten.
